# Autism As a Disorder of High Intelligence

**DOI:** 10.3389/fnins.2016.00300

**Published:** 2016-06-30

**Authors:** Bernard J. Crespi

**Affiliations:** Department of Biological Sciences and Human Evolutionary Studies Program, Simon Fraser UniversityBurnaby, BC, Canada

**Keywords:** intelligence, autism, schizophrenia, genetic correlation, pleiotropy, evolution

## Abstract

A suite of recent studies has reported positive genetic correlations between autism risk and measures of mental ability. These findings indicate that alleles for autism overlap broadly with alleles for high intelligence, which appears paradoxical given that autism is characterized, overall, by below-average IQ. This paradox can be resolved under the hypothesis that autism etiology commonly involves enhanced, but imbalanced, components of intelligence. This hypothesis is supported by convergent evidence showing that autism and high IQ share a diverse set of convergent correlates, including large brain size, fast brain growth, increased sensory and visual-spatial abilities, enhanced synaptic functions, increased attentional focus, high socioeconomic status, more deliberative decision-making, profession and occupational interests in engineering and physical sciences, and high levels of positive assortative mating. These findings help to provide an evolutionary basis to understanding autism risk as underlain in part by dysregulation of intelligence, a core human-specific adaptation. In turn, integration of studies on intelligence with studies of autism should provide novel insights into the neurological and genetic causes of high mental abilities, with important implications for cognitive enhancement, artificial intelligence, the relationship of autism with schizophrenia, and the treatment of both autism and intellectual disability.

## Introduction

‘How wonderful that we have met with a paradox. Now we have some hope of making progress.’

*Niels Bohr*

Autism is conventionally regarded as a neurodevelopmental disorder that involves deficits in social interaction and social communication, combined with restricted or repetitive patterns of behavior and interests. However useful this definition may be for practical purposes, it also represents a reified, more or less arbitrary, historical and societal construction that fits neither with Kanner's (1943) original description (Evans, 2013) nor with the standard medical model of disease whereby maladaptive phenotypes must be understood in terms of alteration to specific adaptive systems (Nesse and Stein, 2012; Crespi, 2016).

Autism may, alternatively, be regarded as a syndrome, a constellation of phenotypes, sets of which tend to be found together relatively often, or sets of which when found together cause particular problems for children, families, and communities (Happé et al., 2006). Any given individual “with” autism will exhibit some more or less unique collection of such phenotypes (e.g., van Os, 2009), which is due to their more or less unique genomic makeup and early developmental environment. By this simple logic, any diagnoses of autism should be regarded not as any sort of endpoint, but as a rough, initial signpost toward eventual determination of the genetic, developmental, hormonal, neurological, psychological, and/or environmental causes of each individual's altered autism-related cognition, affect and behavior.

The finding that autism has many causes (Happé and Ronald, 2008) should direct attention to improved means of differentially diagnosing its personalized bases. This process, in turn, centers on determining what adaptive neural and psychological systems has been altered, and how, to result in some set of autistic traits in some individual. Autistic phenotypes have been linked, for example, to increased protein synthesis at synapses (Bourgeron, 2009), higher excitatory to inhibitory neurotransmission (Rubenstein and Merzenich, 2003), enhanced local compared to global processing and connectivity (Happé and Frith, 2006), a bias toward systemizing over empathizing (Baron-Cohen, 2009), and enhanced perceptual functioning (Mottron et al., 2006). These patterns and theories are not mutually exclusive, but none of them includes an explicit evolutionary dimension, such that their proximate causes remain ungrounded in the one discipline that unites across all biological-psychological levels in the context of ultimate, long-term determinants of psychological adaptation and maladaptation (Crespi, 2016).

Risks for autism, like risks of cancer, diabetes, or arthritis, have evolved along the human lineage (Crespi and Leach, 2016). As such, evolutionary biology becomes a valuable conceptual and analytic tool for connecting adaptive brain systems with the ways in which they can become altered and maladaptive in psychiatric disorders (Fjell et al., 2015). So what adaptive, evolved human brain systems are changed, in what different ways, to produce the phenotypes characteristic of autism? The most prominent neurological change along the human lineage is, of course, the tripling of brain size and associated changes in brain organization and functions, and our concomitant tremendous increase in intelligence compared to other great apes (Roth and Dicke, 2005). A notable set of “brain size genes” has been demonstrated to have been subject to natural selection in humans and other mammals (Montgomery and Mundy, 2014), and variation among extant humans in intelligence is now known to be highly polygenic, underlain by hundreds to thousands of alleles each of small effect (e. g., Davies et al., 2011; Benyamin et al., 2014; Plomin and Deary, 2015).

The genetical evolution of high intelligence in humans has increased scope for two main forms of dysregulation. First, the developmental and neural systems that connect genetic variation and environments with intelligence may be subject to maladaptive alterations by purely deleterious mutations, maladapted genotypes, or harmful environments, that degrade the “intelligence development” system. This route generally leads to what we call intellectual disability (Vissers et al., 2016), with overall reductions in intelligence and the functioning of its physiological and neural subsystems. Second, the development of intelligence can be affected, by genes or environments, in the opposite direction, toward higher levels of functioning. If this change results in balanced enhancements in all of the components of high intellect, general intelligence will be increased. However, if some, or most, but not all inter-dependent general cognitive-intellectual functions are enhanced, what would we observe?

Autism has long been characterized by relatively low intelligence as measured by most standard tests (e. g., Hoekstra et al., 2009). However, a suite of recent studies, described in more detail below, has demonstrated that alleles “for” autism, that is, common alleles that each contributes slightly to its risk, overlap substantially and significantly with alleles “for” high intelligence (Bulik-Sullivan et al., 2015; Clarke et al., 2015; Hill et al., 2015; Hagenaars et al., 2016). To a notable, and well-replicated, degree, then, many “autism” alleles are “high intelligence” alleles. How can these paradoxical observations be reconciled?

In this article I describe and evaluate the hypothesis that a substantial proportion of “autism risk” is underlain by high, but more or less imbalanced, components of intelligence. First, I provide a brief overview of the genetic, developmental and neurological bases and correlates of human intelligence, from research within this particular domain, and relate the structure of intelligence in neurotypical individuals to the differences between autistic individuals and neurotypical individuals. Second, I compare the best-validated correlates of variation among humans in intelligence with established characteristics of autistic individuals, compared to controls. These two areas of study, intelligence and autism, have thus far developed virtually independently from one another; I thus integrate and synthesize them in the context of testing the hypothesis addressed here. In doing so, I also compare results for autism with those for schizophrenia, the other primary human neurodevelopmental disorder, in light of theories for how these two conditions are related to one another (Crespi and Badcock, 2008; Crespi, 2016). Finally, I develop a framework for consilience of these findings with previous theory on autism, and describe the implications of the results, with regard to the causes, treatments, and understanding of autism, and the structure and study of human intelligence. The primary novelty and usefulness of this synthesis is that it provides the first comprehensive connections of the causes and symptoms of autism with alterations to a specific human-elaborated adaptive system, intelligence, and thereby generates new insights and research questions into the natures and inter-relationships of intelligence, autism, and schizophrenia.

## The architecture and correlates of human intelligence

**Figure 1:**
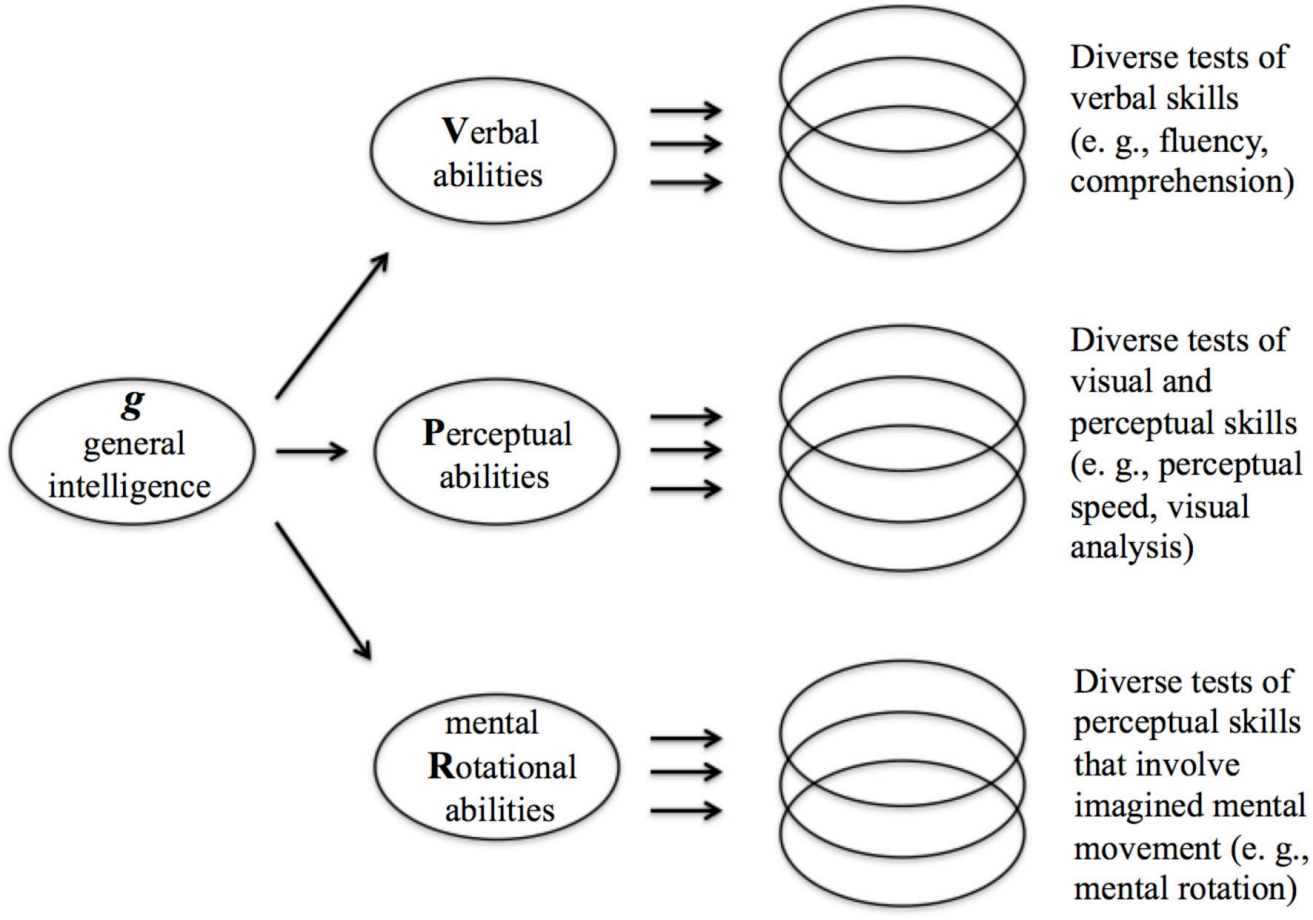
**The VPR model of intelligence**. Under this model, the higher-level architecture of human intelligence, as indicated by diverse mental-ability tasks, involves one general factor, *g*, and three mid-level factors, Verbal, Perceptual, and image Rotation, that reflect variation among individuals in large-scale neural structure and processing. Adapted from Johnson et al. (2007).

Human intelligence has been studied predominantly from psychometric, genetic, neurological, and psychological perspectives. Psychometric studies tracing back to Spearman (1904) have demonstrated that virtually all measures of human mental abilities are moderately to highly positively correlated with one another, such that a common factor, typically called “*g*,” underlies their joint co-variation. Between the general, primary *g* factor, and the diverse, specific measures of mental abilities, is a small set of secondary factors that each statistically accounts for covariation among a larger set of functionally-similar abilities. The Verbal-Perceptual-Rotational (VPR) model of Johnson and Bouchard (2005a,b, 2007; Figure 1), with three such secondary factors, represents the currently best-supported psychometric description of human intelligence structure. Under this model, “Verbal” refers to verbal fluency and knowledge, “Perceptual” refers to perceptual speed and mechanical and spatial abilities other than mental rotation, and “Rotational” refers to mental rotation, which involves mental movements of imagined objects or persons, as in the classic Mental Rotation test from Vandenberg and Kruse (1978; Johnson and Bouchard, 2005b; Major et al., 2012). The components of the VPR model correspond on a broad scale with brain structural organization in that verbal skills are relatively left-hemispheric, spatial and non-verbal abilities are relatively right-hemispheric, and mental rotation depends, in part, on strongly bihemispheric functions associated with corpus callosum size (Johnson and Bouchard, 2005b; Karadi et al., 2006; Schoenemann, 2006).

**Figure 2:**
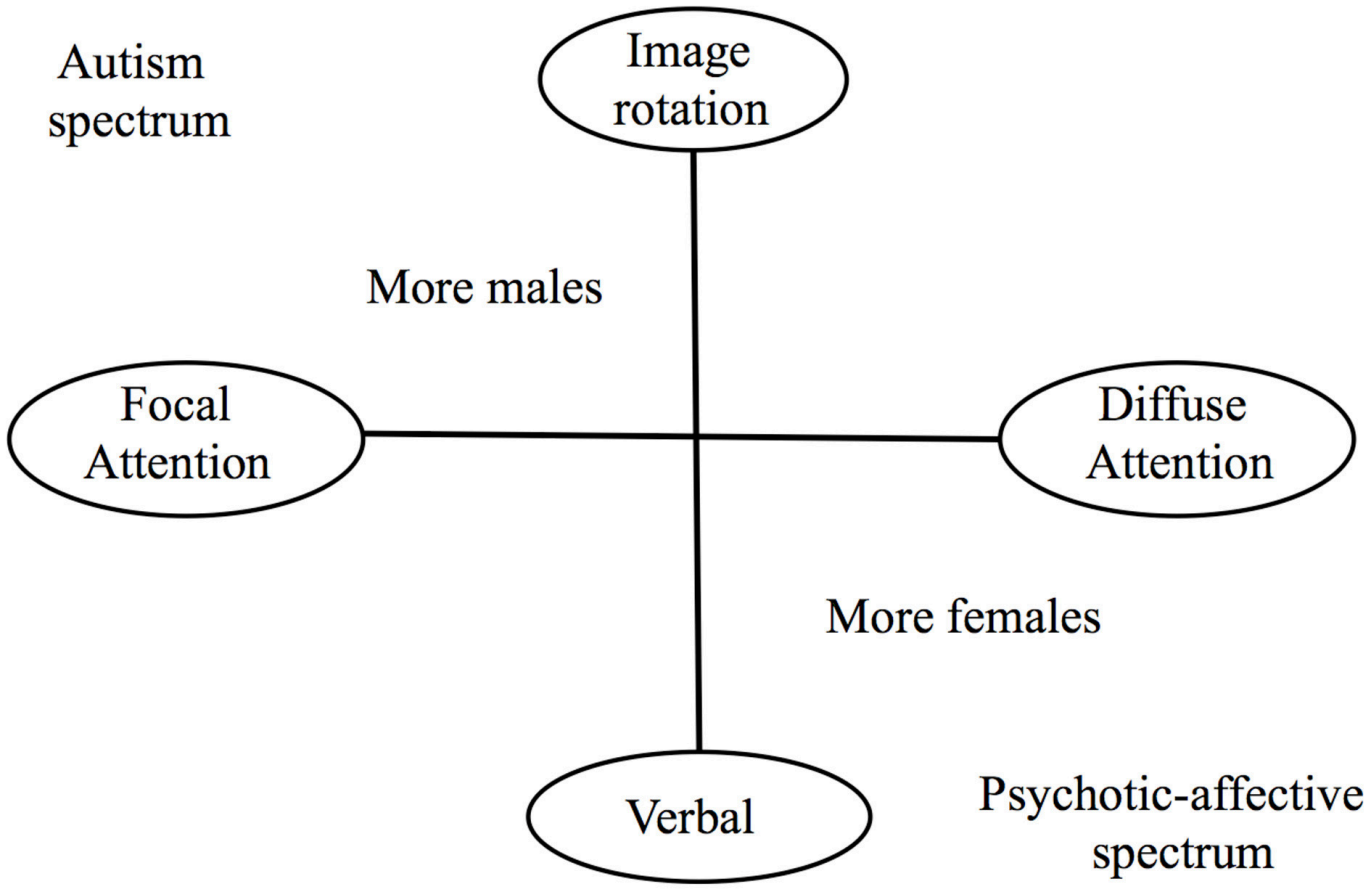
**Two orthogonal dimensions of intelligence, which emerge after the general factor *g* is statistically removed**. Poles of each of the two dimensions demonstrate inverse associations with one another, indicative of neurally-based cognitive trade-offs. The psychotic-affective spectrum includes mainly schizophrenia, bipolar disorder, depression, and borderline personality disorder. Adapted from Johnson et al. (2007).

In addition to its success in describing patterns of co-variation among aspects of human intelligence, the VPR model also demonstrates evidence of tradeoffs between sets of cognitive abilities: when controlling for variation in *g*, image rotation ability is inversely associated with verbal ability, and scores on tests indicative of a strong focus of attention are inversely associated with scores indicating diffuse focus (Johnson and Bouchard, 2007; Figure 2). These two tradeoffs also exhibit sex differences, with males over-represented at the high rotational-strong focus pole, and more females at the pole with higher verbal abilities and more-diffuse focus (Johnson and Bouchard, 2007). Such sex differences and negative correlations are important given the strong male biases found in autism (Fombonne, 2009; Baron-Cohen et al., 2011), the extensive data showing that autism involves reductions in verbal skills but (1) increases in focus of attention (e. g., Ploog, 2010; Sabatos-DeVito et al., 2016), (2) enhanced perceptual and spatial abilities [as reflected in prowess, for example, in Block Design and the Embedded Figures test (EFT); Mottron et al., 2006; Muth et al., 2014], and (3) superior ability in non-rotational (though not rotational) aspects of the mental rotation task (Zapf et al., 2015). Considered together, these findings provide evidence that the cognitive structure of autism dovetails with the structure of the independently derived best-supported model for the psychometric architecture of human intelligence, but that it is characterized by increases in some, specific, components of intelligence and decreases in others, leading to a profile that is imbalanced and reflects extremes of typical variation. As such, autism-related differences in VPR-model structured intelligence appear to reflect its evolutionary bases in altered expression of adaptive cognitive variation (Crespi, 2016), with autistic cognition mediated by relative extremes of tradeoffs (Johnson and Bouchard, 2007, 2008). In contrast to autism, schizophrenia is characterized by a focus of attention decreased relative to controls (e.g., Morris et al., 2013), notably-poor visual-spatial relative to verbal abilities (Kravariti et al., 2006), and reduced ability in non-rotational (though not in rotational) aspects of mental rotation tasks (Thakkar and Park, 2010; Benson and Park, 2013). These patterns of findings derive directly from the VPR model, and indicate that its further application and extension will help to clarify the psychometric structure of autistic, neurotypical, and schizophrenia-associated cognition.

A second model for the higher-level psychometric structure of intelligence is its division into a “fluid” component, reflecting ability to solve novel problems, use logic, and identify patterns, in ways that are independent of acquired and cultural knowledge, and a “crystallized” component, indicative of ability to utilize acquired and learned knowledge and experience (e. g., Nisbett et al., 2012). This subdivision of intelligence fits less well than the VPR model to patterns of covariation across mental ability tests (Johnson and Bouchard, 2005a,b), but its validity and usefulness are supported by the underpinning of fluid vs. crystallized intelligence by distinct sets of genes (Christoforou et al., 2014), their different patterns of change with age (with fluid changing like physical traits, but crystallized showing little age-related decline; Deary et al., 2010), and the primacy of social learning in human cultural adaptation (Henrich, 2015). As for the VPR model, the structure of intelligence in autism reflects the fluid-crystallized dichotomy, in that fluid intelligence is relatively or absolutely enhanced in autism, but crystallized intelligence is reduced, compared to controls (Dawson et al., 2007; Hayashi et al., 2008; see also Nader et al., 2016). This pattern again indicates imbalance, and elevation, in components of intelligence in autism that correspond to its evolved and psychometrically characterized structure.

Intelligence, usually measured by strong correlates of *g*, has a clear polygenic basis, as well as established connections with neurological variation. Recent GWA studies have provided evidence that many hundreds or thousands of alleles, each of very small effect, underlie variation among individuals in *g*, although only a fraction of its high heritability can be accounted for at this point (Plomin and Deary, 2015). By contrast, data from studies of the genetic basis of intellectual disability show that it is due mainly to moderate or large effect *de novo* deleterious alleles, rather than a concentration of weakly-deleterious, small-effect, segregating alleles; these findings indicate that “high intelligence requires that everything work right, including most of the positive alleles and few of the negative alleles associated with intelligence” (Plomin and Deary, 2015, p. 103; Franić et al., 2015; Hill et al., 2015). Plomin and Deary (2015, p. 103) also point out an important question regarding such “positive genetics” of intelligence, for psychiatric disorders: if individuals at the positive end of the polygenic distribution of “risk” simply have low risk, or if they “have special powers.” In this article, I am evaluating the hypothesis that such “special powers” indeed exist, in the contexts of autism and intelligence, and in comparison to schizophrenia. Finally, Plomin and Deary (2015) point out that assortative mating is notably stronger (~0.40) for intelligence than for most other human traits, which maintains additive genetic variation for this trait as well as generating more “extreme” intelligence phenotypes than otherwise expected. Increased autism risk has been attributed by Baron-Cohen et al. (2006) to assortative mating between two individuals high in “systemizing,” and assortative mating is much high among individuals diagnosed with ASD than other disorders (Nordsletten et al., 2016); how might intelligence variation play a role in this process and its sequelae?

**Figure 3:**
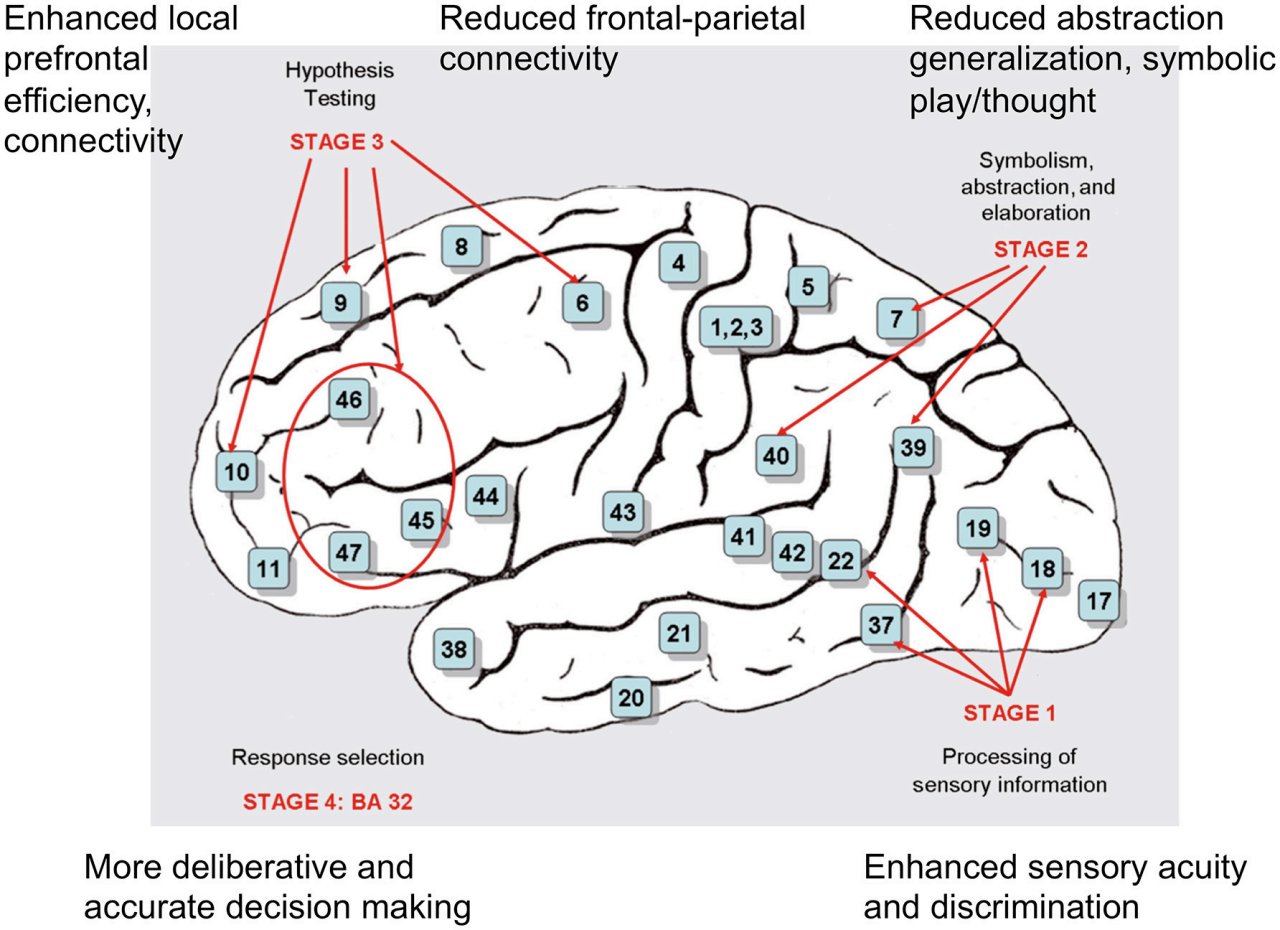
**Brain regions, and temporal stages, representing the P-FIT (Parietal Frontal Integration Theory) model of intelligence**. Postulated alterations in autism, compared to control individuals, are shown along the periphery, and described in the text. Adapted from Colom et al. (2010).

As described in detail below, the neurological basis of intelligence has been well established for a suite of phenotypes, including large brain size and high numbers of neurons, large hippocampus size, high efficacy of working memory, fast neuronal processing speed, neural efficiency, fast rates of brain growth and pruning, and specific patterns of gray matter and white matter distributions. Most broadly, high intelligence appears to reflect high functionality, speed, and integration of a fronto-parietal brain network that subserves the sensory acquisition, abstraction, alternative model-testing, and deployment of information (the Parieto-Frontal Integration Theory; Jung and Haier, 2007; Colom et al., 2010; Figure 3). This “intelligence network” overlaps substantially with the “task-positive network,” whose activation is inversely associated with that of the “default mode” or “task-negative” network (e.g., Uddin et al., 2009), as might be expected given that mental abilities and intelligence are measured in the context of particular tasks. The general and specific functioning of this distributed, fronto-parietal network are highly compatible with the VPR psychometric model described above (Deary et al., 2010), with genetic bases to VPR abilities and fronto-parietal structure and function (Johnson et al., 2007).

In addition to its substantial psychometric and genetic basis, and neurological underpinnings, intelligence is also notably associated with two additional factors that are relevant to the autism spectrum: sensory abilities and socioeconomic status. Positive correlations of *g* with sensory abilities in the auditory, visual, and tactile domains have been well documented and replicated, across over a century of testing (Deary et al., 2004). Such sensory tests include, for example, ability to discriminate closely-similar stimuli, temporal information processing skill, or sensitivity in stimulus detection, that apparently reflect aspects of processing speed, focal-attentional abilities, and ability to suppress irrelevant stimuli. As described further below, Galton (1883) and Spearman (1904, pp. 268–272) indeed virtually equated higher cognitive abilities with more-accurate sensory discrimination, and postulated “general discrimination” skills that underpinned sensory abilities across different modalities and correlated near unity with general intelligence. Genetically-based positive associations of intelligence with socioeconomic status, as well as with education levels and occupational status, have also been well documented (Marioni et al., 2014; Trzaskowski et al., 2014; Krapohl and Plomin, 2016; review in Plomin and Deary, 2015). These findings indicate that the same alleles pleiotropically mediate high intelligence and high socioeconomic status (vs. low levels of both), again through many alleles of small effect that account for a small, though statistically significant, proportion of the variance in both traits.

The upshot of these considerations from psychometrics, genetics, neuroscience, psychology, and sociology is that high intelligence involves many beneficial alleles of small effect, an absence of *de novo* deleterious mutations, high performance with balanced integration of neural subsystems (as well as large brain size and more neurons), enhanced sensory abilities, high socioeconomic status, and a notable degree of assortative mating. How, then, do these genetic and phenotypic correlates of intelligence, and associated ones, relate to the genetic basis and phenotypic correlates of autism?

## Genetic overlap of autism with intelligence

Recent increases in sample sizes, extensions of target phenotypes analyzed, and developments in analytic methods for genome-wide association studies, have allowed the first robust tests of the sign and magnitude of genetic correlations, due to pleiotropy and linkage disequilibrium, between intelligence and other traits including risk of psychiatric conditions. Four studies have used data from the Psychiatric Genetics Consortium (PGC) on polygenic risk for autism (Cross-Disorder Group of the Psychiatric Genomics Consortium, 2013) to assess overlap of autism risk alleles (identified from over 5000 cases) with alleles for aspects of cognitive ability and intelligence. All four of these studies, which used diverse, independent populations and tests (or correlates) of cognitive abilities, have reported significant, substantial genetically-based positive associations of autism risk with intelligence, notably including full-scale IQ and a PCA-based measure of *g* (Clarke et al., 2015), childhood IQ, college attendance, and years of education (Bulik-Sullivan et al., 2015), cognitive function in childhood and educational attainment (Hill et al., 2015), and verbal-numerical reasoning and educational level reached (Hagenaars et al., 2016). These studies indicate that polygenic, small-effect size alleles that increased risk of autism are also associated with increased intelligence (and strong correlates of intelligence, such as education level; Davies et al., 2016) among neurotypical individuals.

In direct contrast to these findings for autism, genetic risk for schizophrenia has been demonstrated to be negatively associated with measures of cognitive ability and intelligence, across a broad suite of studies (McIntosh et al., 2013; Lencz et al., 2014; Bulik-Sullivan et al., 2015; Hill et al., 2015; Hagenaars et al., 2016; Hubbard et al., 2016). These results indicate that a substantial proportion of schizophrenia risk alleles also represent alleles “for” lower intelligence. Such findings for schizophrenia accord well with large bodies of research on cognitive deficits in first-degree relatives of individuals with schizophrenia (Snitz et al., 2006), and among children who are premorbid for schizophrenia (and thus will develop it at or after adolescence; Woodberry et al., 2008).

The question of whether intelligence, or any of its components, are elevated, compared to controls, among parents, siblings, or higher-order relatives of individuals with autism has yet to be investigated systematically, although this pattern is predicted by the positive genetic correlations of autism risk with measures of IQ. Tests conducted thus far have yielded diverse results, but individuals with autism clearly exhibit a similar cognitive profile to their sibs across WAIS subtests, with high scores on Block Design and Object Assembly, and low scores on Comprehension and Coding, relative to controls (Gizzonio et al., 2014). Similarly, siblings of individuals with autism have shown better working memory (for non-social targets) than control individuals (Noland et al., 2010) and higher visual-motion sensitivity (McCleery et al., 2007), and parents of individuals with autism are faster than controls at the EFT (Baron-Cohen and Hammer, 1997). Kanner (1943) and Rimland (1964, pp. 29–30) believed that parents of individuals with autism were of higher intelligence than control parents (see Levine and Olson, 1968), but their hypotheses have apparently not been subject to robust empirical testing, with most such studies of parents focusing on social deficits and alterations. By contrast, as described above, there is clear evidence for relatively low IQ, on average, among individuals with autism, at least as measured by most standardized tests.

How can this paradox of low IQ, but positive genetic correlations of autism risk with intelligence, be resolved? None of the papers on genetic correlations of autism with intelligence discuss possible explanations, or ways to investigate the conundrum further. I have proposed here the hypothesis that autism involves high but imbalanced intelligence, such that some or many genetically-based components of intelligence are enhanced, but imbalance across components increases risk and patterns of expression for autistic phenotypes, and for diagnoses. By this hypothesis, higher intelligence may co-occur with higher risk for imbalance and cognitive and affective consequences from it, given that the components of cognitive ability are expected to interact strongly and may do so to a greater degree at the higher end of ability. The “high intelligence imbalance” hypothesis is useful because it makes clear predictions, and thus directs attention toward specific forms of existing data and new, informative future data to collect.

## Autism and the correlates of intelligence

The “high intelligence imbalance” hypothesis predicts that autism should be associated, at a phenotypic level, with substantiated correlates of intelligence. I elaborate here on the most-notable joint correlates of intelligence and autism, focusing on phenotypes that are associated with intelligence and that are over-developed or over-expressed in autism.

### Brain size and growth

Large brain size and head circumference, especially in childhood but also adulthood, represent some of the best-substantiated phenotypic correlates of autism (e.g., Fukumoto et al., 2011; Foster et al., 2015; meta-analysis in Sacco et al., 2015). Autism-linked increases in brain size have been shown to involve higher numbers of neurons (Courchesne et al., 2013), a thicker cortex (Hardan et al., 2006; Karama et al., 2011; Ecker et al., 2013; Smith et al., 2016), increased hippocampus volume (Barnea-Goraly et al., 2014; Maier et al., 2015), increased brain growth rates in early childhood (Campbell et al., 2014), increased rate of cortical thinning in adolescence (Hardan et al., 2009; Mak-Fan et al., 2012), a combination of “accelerated expansion in early childhood” with “accelerated thinning in in later childhood and adolescence” (Zielinski et al., 2014), and increased processing of more-local, detailed information (White et al., 2009).

Faster increase in cortical thickness between ages 6 and 12, followed by faster cortical thickness deceleration between ages 12 and 18 (indicative of neuronal and synaptic pruning), has been linked with higher intelligence in typically-developing children (Shaw et al., 2006). These findings provide evidence that trajectories of brain growth rate during middle childhood to adolescence are notably associated with IQ, with an overall pattern of accelerated growth and accelerated pruning that matches trajectories reported in autism, though with different timings of growth in early childhood. Within humans (e.g., Ivanovic et al., 2004; Witelson et al., 2006; Menary et al., 2013), and among non-human primates (Deaner et al., 2007) species, brain size (and cortical thickness, for humans) are also positively correlated with measures of intelligence, an effect that appears to be mediated predominantly by numbers of neurons (Roth and Dicke, 2005; Dicke and Roth, 2016).

In contrast to these patterns in autism, brain size, hippocampus size, and cortical thickness are reduced among individuals with schizophrenia, including at first episode (e.g., Steen et al., 2006; Rais et al., 2012; Rimol et al., 2012; Oertel-Knöchel et al., 2013). These reductions appear to be associated with reduced brain growth rates in late childhood and early adolescence (Gogtay et al., 2008), followed by increased gray matter loss in adolescence and early adulthood (e.g., Pantelis et al., 2005; Rapoport and Gogtay, 2011).

The genetic bases of brain size and growth, and intelligence, in relation to autism and schizophrenia, remain largely unknown. However, as one example of such inter-relations, numbers of repeat units of a protein domain referred to as *DUF1220* have been positively associated with a suite of characters including: (1) brain size (within humans, and across species of anthropoid primates; Dumas et al., 2012; Keeney et al., 2014), (2) IQ and mathematical aptitude (Davis et al., 2015), and (3) the severity of autism (Davis et al., 2014); by contrast, *DUF1220* repeat numbers are inversely associated with positive-symptom (though not negative-symptom) severity in schizophrenia (Quick et al., 2015). These findings indicate strongly pleiotropic effects of this molecular domain on brain size, intelligence, autism, and schizophrenia, whereby autism is linked with large brain size and high IQ, but positive symptom schizophrenia shows a diametric result.

### Brain connectivity

Low global, relative to local, structural and functional connectivity of the brain has been demonstrated in autism by a large suite of studies (reviews in Courchesne and Pierce, 2005; Maximo et al., 2014), and it follows in part, presumably, from increased brain size itself. Under the P-FIT model of intelligence (Figure 3), effective long-range connectivity is required for the integration of parietal and frontal brain regions that underlies IQ (Jung and Haier, 2007); “efficient” patterns of brain connectivity involving small-world network-level organization (with an optimal mix of short and long-rage connections) have thus, for example, been positively associated with measures of intelligence (van den Heuvel et al., 2009; Koenis et al., 2015; Kim et al., 2016). These findings suggest that relative reductions in long-range connectivity may represent an important constraint on general intelligence among individuals with autism, contributing to imbalances between its constituent parts. Testing of this hypothesis will, however, require analyses of local and global brain connectivity patterns in relation to both autism (compared to controls) and variation in intelligence (in each group) using the same methodology, to determine the degree to which the long-range connectivity pathways central to the P-FIT model (e.g., the arcuate fasciculus, and connections to the lateral prefrontal cortex; Jung and Haier, 2007; Cole et al., 2012) are differentially reduced in efficiency among individuals with autism.

In contrast to reduced long-range brain connectivity, increased local connectivity has been linked with enhanced ability in some domains, such as auditory pitch perception (Loui et al., 2011), which shows a notable association with the autism spectrum (Stanutz et al., 2014). Increased local connectivity, especially of the prefrontal cortex, also characterizes the valproic acid animal model of autism (Rinaldi et al., 2008) and the “intense world” theory of autism etiology (Markram and Markram, 2010), which involves perception, attention, and memory that are enhanced to levels that interfere with social functioning.

Higher local connectivity in sensory regions of the brain has been suggested as the basis for sensory hyper-sensitivities in autism (Belmonte et al., 2004) as well as hyper-developed attention to detail and systemizing (Baron-Cohen et al., 2009). Considered together, these findings suggest that increased local brain connectivity in autism is linked with specific enhanced abilities or interests, such that components or facets of general intelligence are increased whereas *g* itself is reduced. These brain network alterations in autism can be described most simply as involving increased brain modularity and parallel processing, which may enhance region-specific functions (such as sensory abilities and visual-spatial skills) but also lead to reduced general intelligence due to under-developed long-range connectivities.

Decreased local and increased long-range connectivity have been described in childhood-onset schizophrenia (Baribeau and Anagnostou, 2013), and increased connectivity has also been found within the default mode in schizophrenia across multiple studies (Whitfield-Gabrieli et al., 2009; Tang et al., 2013), with several reviews pointing out the opposite nature of this pattern compared to that found in autism (Broyd et al., 2009; Karbasforoushan and Woodward, 2012). However, robust tests of this hypothesis require joint connectivity analysis of individuals with autism and schizophrenia using the same protocols.

### Neuronal function

Synaptic plasticity represents a core component of brain function, and, in principle, it underlies on a neuronal scale the long-term macroscopic changes in cortical thickness across childhood and adolescence that have been linked with intelligence (e.g., Shaw et al., 2006). Protein synthesis in dendritic spines mediates synaptic plasticity, and has been associated with diverse aspects of cognition, learning and memory (Sutton and Schuman, 2006; Kasai et al., 2010). Evidence of exaggerated protein synthesis at dendrites has been reported in human syndromic autism and in multiple animal models of autism (Kelleher and Bear, 2008; Bourgeron, 2009; Gkogkas et al., 2013; Santini et al., 2013; Santini and Klann, 2014; review in Mottron et al., 2014), indicating that gains of function or expression in key molecular mediators of cognition may characterize the autism spectrum (e.g., Figure 1 in Kulkarni and Firestein, 2012). Increased levels of neuronal plasticity and synaptic remodeling likewise characterize some theory for autism and animal models (Markram and Markram, 2010; Isshiki et al., 2014; Oberman and Pascual-Leone, 2014).

The degree to which neuronal functions such as synaptic plasticity, dendritic spine protein synthesis levels, and dendrite dynamics including flexibility and stability influence variation in general intelligence remains unclear. However, a recent GWA study found that the strongest functional enrichment for genes linked with fluid intelligence was synaptic “efficiency,” whereas for crystallized intelligence it was synaptic depression and LTD (long-term depression; Christoforou et al., 2014). These findings suggest strong associations of neuronal and synaptic function with intelligence, such that autism may commonly involve dysregulation of intelligence-associated neuronal processes toward hyper-functional dynamics. The gene *CYFIP1* represents an apparent example of a locus that mediates such effects: high expression of this gene, which coordinates mRNA translation at dendrites, has been associated with autism (Oguro-Ando et al., 2015; Wang et al., 2015); by contrast, deletions that reduce its expression have been strongly linked with risk of schizophrenia, as well as with aspects of impaired cognition (especially dyslexia and dyscalculia) among otherwise-neurotypical individuals (Stefansson et al., 2008, 2014; Tam et al., 2010).

### Sensory functions, attention, and special abilities

As noted above, Galton (1883) and Spearman (1904) first described hypotheses and psychometric evidence that sensory abilities and sensory discrimination skills were strongly positively associated with high intelligence. A resurgence of interest in this phenomenon has led to consistent and diverse evidence for small to moderate links of specific sensory discrimination abilities with intelligence, but strong correlations (e.g., 0.68 and 0.92 in Deary et al., 2004) of intelligence with latent factors that integrate sensory ability variation across domains (Meyer et al., 2010). The causes of correlations between general intelligence and sensory discrimination abilities remain largely unknown, but they appear to be related to: (a) ability to focus intensely while ignoring irrelevant stimuli (Melnick et al., 2013); (b) a strong positive genetic correlation between intelligence and sensory-neural processing speed (Lee et al., 2012); (c) speed of neural oscillations, which may underlie both sensory discrimination skills and intelligence (Troche and Rammsayer, 2009a,b; Troche et al., 2009); (d) white matter structure and integrity, which are positively associated with neural processing speed (e.g., Turken et al., 2008; Kerchner et al., 2012); (e) regional or global increases in gray matter (e.g., Deary et al., 2010; Hyde et al., 2010), and (f) the role of sensory input as a limiting step in general cognitive ability, upon which all further neurological components of intelligence depend. Further evaluation of these hypotheses, through GWAS-based tests for genetic correlation and neurological studies that jointly address sensory discrimination and intelligence, are required to evaluate their robustness and generality.

A large body of evidence has shown that sensory discrimination and sensory acuity abilities are commonly enhanced in autism compared to controls, across auditory (O'Riordan and Passetti, 2006; Heaton et al., 2008; Eigsti and Fein, 2013; Stanutz et al., 2014), visual (Ashwin et al., 2009; Brosnan et al., 2012; Falter et al., 2012), and tactile (Blakemore et al., 2006; Cascio et al., 2008; Nakano et al., 2012) domains. As for the links with IQ, causation remains largely obscure. However, Blaser et al. (2014) demonstrate that the autism-associated advantage in visual search is associated with stronger phasic pupillary response, which is indicative of stronger attentional focus and implicates the locus coeruleus-norepinephrine system in (at least) visual discrimination proficiency. This finding is of especially notable interest given that autism is characterized, on a general diagnostic basis, by increased attention to detail, difficulties in switching of attention, and attentional stimulus “overselectivity” on specific aspects of the physical environment (Murray et al., 2005; Ploog, 2010). High attention to detail on the Autism Quotient test, high intelligence, and high rates of autism in family members have also been reported among child prodigies (children who display highly-advanced abilities in fields such as music, mathematics, chess, or art; Ruthsatz and Urbach, 2012). Baron-Cohen et al. (2009) describe evidence that such high attention to detail in autism is a consequence of enhanced sensory abilities, and also leads to high levels of an analytical, “systemizing” cognition. Finally, Sabatos-DeVito et al. (2016) describe experiments that link atypical sensory processing in autism to attentional engagement, suggesting that these two facets of autism share neurological and psychological links.

One visual-spatial test, the embedded-figures test (EFT), represents a paradigmatic task showing superiority in autism for speed, accuracy, or both (Happé and Frith, 2006; Muth et al., 2014; Horlin et al., 2016). This test has traditionally been considered as indicative of a local cognitive “style,” but three sets of findings: (a) high positive correlations of EFT performance with measures of fluid intelligence (e, g., McKenna et al., 1986; McKenna, 1990), (b) demonstration by Khodadady and Tafaghodi (2013) that EFT performance is strongly, positively linked with intelligence, and (c) the visual-spatial search and discrimination nature of the task, suggest that it can also be interpreted as a metric of visual sensory ability and discrimination that is, like other such metrics, highly associated with intelligence, especially fluid intelligence that is often not matched in autism-control comparison studies. In contrast to the patterns of EFT enhancement found for autism, meta-analysis indicates that EFT performance is significantly and substantially reduced in schizophrenia (Panton et al., 2016). Indeed, more generally, sensory discrimination and abilities are consistently reduced in schizophrenia (Bates, 2005; Force et al., 2008; Javitt, 2009a,b), as expected under the hypothesis that they represent psychologically-diametric conditions (Crespi and Badcock, 2008; Crespi, 2016).

Considered together, these findings suggest that increased sensory discrimination ability in autism represents a component or strong correlate of intelligence that is frequently enhanced to the point of imbalance with other aspects of IQ. Indeed, under the P-FIT model (Figure 3), sensory abilities are represented by the first, sensory processing stage of intelligence circuitry: data acquisition and coding mainly via occipital and parietal regions of the brain. Hyper-functioning of these regions may thus result in imbalanced intelligence, whereby efficient integration with downstream regions, especially parietal regions that subserve symbolism, abstraction and categorization of sensory information, becomes dysregulated (e.g., Froehlich et al., 2012; Church et al., 2015; Eduardo Mercado et al., 2015; Figure 3). The gene *GABRB3*, which codes for a GABA receptor, represents a possible example of a locus that is pleiotropically linked to sensory sensitivity, autism risk, and components of intelligence, given that SNPs in this gene have been linked with tactile sensitivity, risk of Asperger syndrome, and scores on the embedded figures and mental rotation tests (Tavassoli et al., 2012; Warrier et al., 2013).

Finally, autism is the only psychiatric condition characterized by notable rates of savant skills, which in this context represent highly-structured, rule-based abilities largely restricted to a few spheres of mental ability: calendar calculating, rote memory, mathematical computation, musical memory, and realistic drawing (Howlin et al., 2009; Snyder, 2009; Treffert, 2014; Meilleur et al., 2015). Savantism appears to represent an extreme of imbalanced components of mental ability in autism, given its highly limited range of enhancements and apparent negative associations of special skills with verbal and social abilities (Crespi and Leach, 2016).

### Decision-making

Decision making, or “response selection,” mediated by the anterior cingulate cortex, represents the fourth stage in the P-FIT model of intelligence (Jung and Haier, 2007; Figure 3). Autism has been characterized, in a recent suite of studies, by more “deliberative” decision making (compared to controls), that tends to reduce biases and errors associated with fast and intuitive, but often “irrational,” decision-making (De Martino et al., 2008; Brosnan et al., 2014, 2016; South et al., 2014). Given that susceptibilities to cognitive biases are negatively associated with measures of intelligence, weakly though significantly (e.g., Teovanović et al., 2015), these findings suggest that this component of intelligence is enhanced in autism, at least in some contexts. More-deliberative decision making in autism may be associated, and underpinned, by enhanced explanatory drive to seek information in ambiguous circumstances, with regard to physical (rather than social) problems (Rutherford and Subiaul, 2015).

In contrast to these results for autism, some cognitive biases, such as “jumping to conclusions,” and “bias against disconfirmatory evidence” are increased in schizophrenia compared to controls (Woodward et al., 2006; Dudley et al., 2016). Similarly, performance on the Iowa Gambling task of decision-making ability is reduced in schizophrenia (Sevy et al., 2007; Adida et al., 2011) but increased in autism (South et al., 2014), in each case compared to controls. Despite such findings, the degree to which more-deliberative or enhanced decision-making is more intelligent *per se*, and does not itself entail costs, remains unclear; for example, faster, more-intuitive decision-making may be favored in many social situations (South et al., 2014), and rationality appears to be at least partially dissociable from intelligence (Stanovich and West, 2014).

### Socioeconomic status

Socioeconomic status, intelligence, and education level achieved have been demonstrated to exhibit strong positive correlations amongst themselves, although the reasons for these associations have remained unspecified (Deary and Johnson, 2010). A recent suite of studies has shown that such links are substantially genetically based, indicating that a large number of alleles pleiotropically affect socioeconomic status, intelligence, and educational achievement (Marioni et al., 2014; Trzaskowski et al., 2014; Krapohl and Plomin, 2016). These findings are of interest in the context of autism because autism also shows significant, positive genetic correlations with educational attainment, which is strongly positively associated with socioeconomic status as well (Bulik-Sullivan et al., 2015; Hill et al., 2015; Hagenaars et al., 2016).

The hypothesis that autism risk in offspring is positively associated with high parental intelligence, and high socioeconomic status, traces to Kanner (1943, p. 248), who stated, referring to autistic children, that “they all come of highly intelligent families,” at high levels of educational, socioeconomic and occupational achievement (Kanner and Lesser, 1958; Rimland, 1964). King (1975) reviewed a set of demographic studies motivated by these findings, and reported strong support for the pattern of high socioeconomic status linked with autism, including support from studies (e.g., Lotter, 1966, 1967) that checked all young children (of 8–10 years) in a given geographic area for infantile autism, and thus should be largely independent of confounding ascertainment or help-seeking biases, variation in access to relevant health care, or variation in parental awareness.

Recent studies of socioeconomic correlates of autism have generated variable results, most of which, however, find that autism is positively related to indicators or strong correlates of high socioeconomic status (Durkin et al., 2010; Van Meter et al., 2010; King and Bearman, 2011; Leonard et al., 2011; Thomas et al., 2012; Bakian et al., 2015); other studies report links with low socioeconomic conditions, or no associations (Rai et al., 2012; Sun et al., 2014). The degree to which biases and confounding factors mediate the positive associations remains largely unknown, although it is noteworthy that both mild to moderate intellectual disability, and schizophrenia, show notable patterns of association with measures of low socioeconomic status (Werner et al., 2007; Leonard et al., 2011; Emerson, 2012; Zheng et al., 2012).

Given that autism risk shows strong genetic correlations with intelligence and years of education, and that these two variables are strongly linked with higher socioeconomic status in a demographic context, the findings described above suggest that autism risk and high socioeconomic status are also expected to show a basis in pleiotropy as demonstrated by positive genetic correlation. However, this hypothesis requires direct genetic tests that take account of known confounding factors and inter-correlated traits (King and Bearman, 2011), as well as analyzing hypotheses for the causal underpinnings of the genetic and phenotypic associations.

### Profession

Profession, occupation, and vocational interests have been associated with autism in the context of Baron-Cohen's theory that the autism spectrum is mediated by high “systemizing” (drive to understand non-social, mechanistic and rule-based systems) combined with low “empathizing” (drive to understand and connect with people, socially and emotionally; Baron-Cohen, 2009). Under this systemizing-empathizing theory, persons expressing high levels of autism spectrum psychological traits are predicted to engage in, or plan to enter, professions that involve systemizing, especially engineering and the physical, mathematical and technical sciences. This prediction of the theory has received considerable, though not fully unequivocal, support (Baron-Cohen et al., 1997, 1998, 2007; Windham et al., 2009; Campbell and Wang, 2012; Roelfsema et al., 2012; Spek and Velderman, 2013; Wei et al., 2013). In contrast to these results, schizophrenia and mood disorders are associated with professions in the arts and humanities, across a diverse array of studies (Nettle, 2006; Kyaga et al., 2011; Campbell and Wang, 2012; Crespi et al., 2016).

Associations of autism with technical professions, in the context of autism's links with intelligence, raise the issue of whether intelligence, as measured by tests of IQ, varies in relation to profession. This controversial topic has been addressed in a suite of studies, all of which report that more-technical professions or occupational plans, especially in engineering, the physical sciences, and mathematics, are associated with relatively high IQs or strong correlates of IQ (Wolfle and Oxtoby, 1952; Hauser, 2002; Wai et al., 2009; Eysenck, 2012 p. xi). The psychological, sociological, and economic causes of these findings are ambiguous, but the results, taken at face value and in conjunction with the vocational correlates of autism, support the hypothesis that the autism spectrum is associated in some manner with relatively high intelligence.

In the context of Baron-Cohen's systemizing-empathizing hypothesis, these findings appear somewhat problematic, because systemizing, as measured by the Systemizing Quotient questionnaire, appears to be uncorrelated with IQ (Ling et al., 2009). Given the diversity of causes of autism, this condition may, however, certainly be associated with both systemizing and high, imbalanced intelligence, even if the two are not connected in simple or direct and causal ways.

### Assortative mating

Positive assortative mating, the mating between individuals who are relatively-similar for a given phenotype or genotype, results in a disproportionate concentration of the relevant alleles among offspring, an increase in additive genetic variance for the trait, and a concomitant rise in heritability (Plomin and Deary, 2015). Humans mate positively assortatively for a wide variety of phenotypes, with intelligence as one of the traits exhibiting the highest correlation between mates, on the order of 0.40–0.60 (Escorial and Martín-Buro, 2012; Plomin and Deary, 2015). To the extent that high intelligence potentiates imbalance in the components of intelligence due to either increases or reductions in its parts, strong assortative mating for intelligence is expected to intensify its effects.

Is there also assortative mating for autism or autism spectrum traits? Baron-Cohen et al. (2006) describe evidence of assortative mating between couples who are both high in the autism-associated psychological trait of systemizing. This hypothesis is supported, for example, by findings that both fathers and mothers of children with ASD exhibit elevated rates of systemizing-related occupations in their fathers (Baron-Cohen et al., 1997), as well as both showing high performance on the EFT (Baron-Cohen and Hammer, 1997). The most direct evidence for assortative mating for autistic phenotypes comes from Nordsletten et al. (2016), who reported much higher rates of assortative mating by psychiatric diagnosis for adults with ASDs (0.45–0.48), than for any other of a large set of disorders. To the extent that autism is genetically correlated with metrics of high intelligence (as described above), these findings indicate that humans mate positively assortatively not just for intelligence, but also for the autism-associated genetic underpinnings of intelligence. Genetic consequences for offspring would thus include both high intelligence and elevated risk of autism, provided that, under the intelligence-imbalance hypothesis addressed here, this process also involved dysregulation of one or more of its components. Further evaluation of this hypothesis would benefit from determining the degree to which positive assortative mating also occurs for autism-related cognitive phenotypes in non-clinical populations, data which would indicate the generality and strength of any such effects.

## Discussion

Risk and expression of autism is mediated by alterations to adaptive, evolved cognitive systems, and human intelligence represents one of the most important and pervasive changes along the human lineage and a principal source of cognitive variation among individuals. In this article, I have described the novel paradox that autism is positively genetically correlated with high intelligence, even though individuals with autism tend to have substantially lower IQs than controls. I then evaluated the idea that the paradox can be resolved under the hypothesis that autism involves high yet imbalanced intelligence, such that some or most components of intelligence are increased, but in such a way that overall performance is often reduced. This hypothesis extends previous studies of intelligence in relation to autism (e.g., Dawson et al., 2007; Hayashi et al., 2008; Nader et al., 2016) by providing the first comprehensive integration of the study of intelligence with the study of this condition, in the context of a novel “high and imbalanced intelligence” model that provides specific predictions and guidance for future work. The primary conclusions and implications from testing the hypothesis are four-fold.

First, the psychometric structure of human intelligence, as encompassed by the VPR model and the fluid/crystallized dichotomy, corresponds well with the differences in cognitive profiles between individual with autism and controls. Autism thus involves absolutely or relatively enhanced abilities in the Perceptual domain, but reduced or preserved Verbal and Rotation skills, and absolutely or relatively enhanced fluid intelligence, but reduced or preserved crystallized intelligence. Given that Perceptual domain tasks and tests quantify visual-spatial, sensory discrimination, mechanistic, scientific, and attentional abilities and motivations (Johnson and Deary, 2011), such enhancements are consistent with a large body of previous work on autism but can serve to unify and connect such skills with their neurological and genetic bases. The VPR model can also help to explain the male bias in autism as related to increased focus of attention, reduced verbal skills, and enhanced image rotation ability (or components thereof), given that these patterns emerge from the VPR model once the effects of *g* are controlled. Considered together, these results imply that although major aspects of intelligence differ between individuals with autism and controls, the differences align with the evolved, neurologically-based axes of cognitive architecture that underlie human mental abilities. Finally, this model may help to frame hypotheses for the autism-related co-variation in perceptual abilities described by Meilleur et al. (2014), perhaps as a manifestation of the increased importance of this facet of intelligence in autistic cognition.

The main implications of these results are that they provide a non-arbitrary, well-validated context (the theory of intelligence) for the interpretation of differences between individuals with and without autism, and they should motivate novel and comprehensive integration of the study of intelligence with the study of autism. With regard to treatments for autism, such integration is useful because it indicates that imbalances in components of intelligence, and their neural underpinnings, may represent novel and malleable targets for individualized therapies that seek to increase the degree of balance, thereby reducing autism symptoms and enhancing everyday social and non-social functioning and well-being. In both phenotypic and genetic contexts, future studies of intelligence in autism might usefully focus on individuals with autism that is apparently mediated by polygenic (rather than monogenic, oligogenic, or syndromic) effects, given that only such causes of autism are expected to be directly relevant to its positive genetic correlation with intelligence. Do such individuals have “too many,” or a biased neurological-associated set, of alleles for high intelligence? What developmental and molecular pathways are affected by such sets of genes, and can they also serve as foci for therapies?

**Figure 4:**
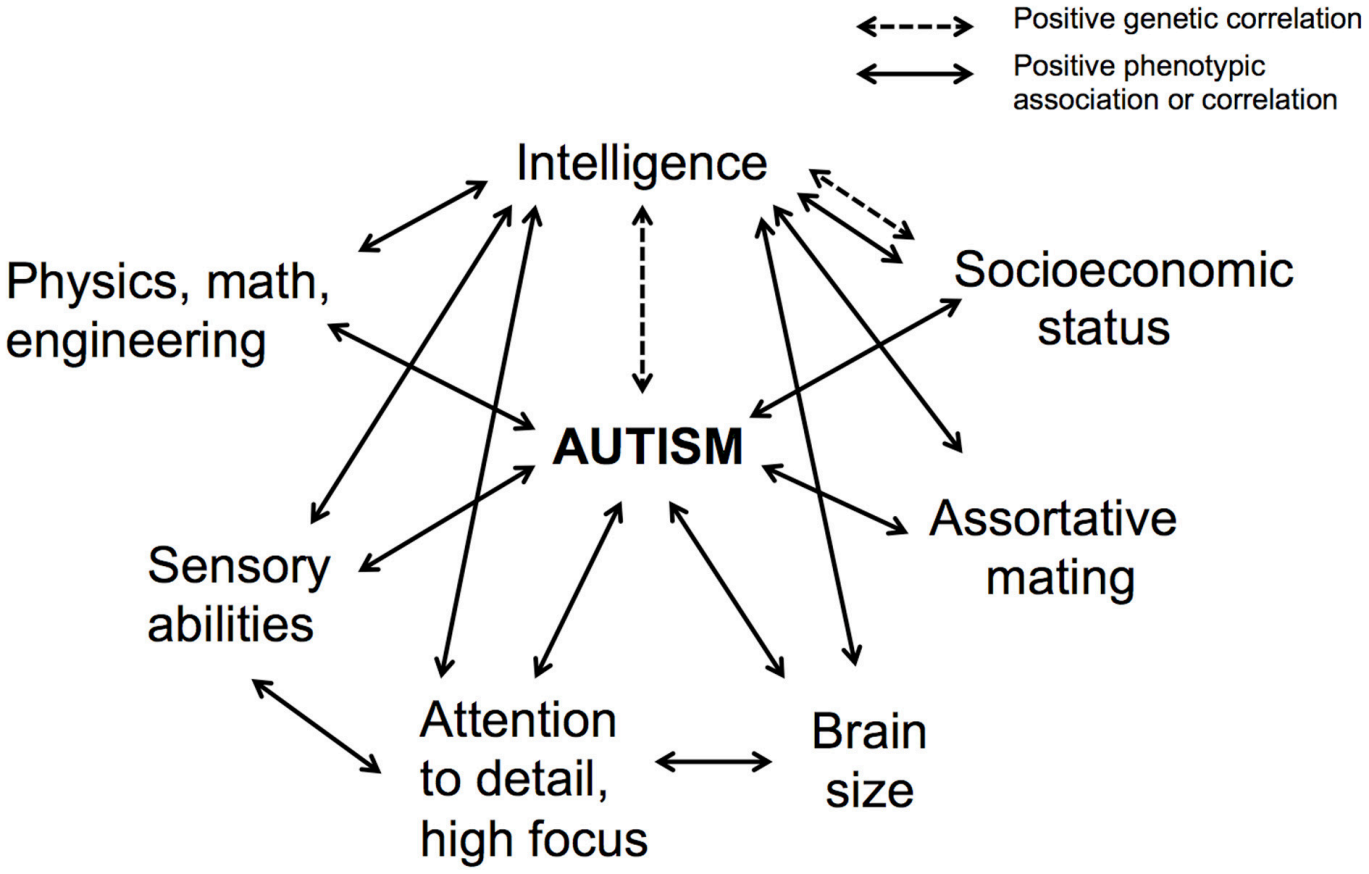
**Autism and intelligence are genetically correlated with one another, indicative of a shared genetic basis, and they share phenotypic correlations or associations with a broad suite of traits**. These patterns suggest that autism risk is mediated in part by high, but imbalanced, components of intelligence.

Second, a broad swath of correlates of autism, including large brain size, fast brain growth, increased sensory and visual-spatial abilities, enhanced synaptic functions, increased attentional focus, high socioeconomic status, more deliberative decision-making, profession and occupational interests in engineering and physical sciences, and high levels of positive assortative mating, also represent strong correlates of intelligence (Figure 4). These findings broadly support the high, imbalanced intelligence hypothesis, although targeted tests are required for more-robust evaluation. Future studies can usefully focus on how these joint correlates of autism and intelligence are related to one another, especially across levels from genes to neurobiology and psychological traits.

Third, the theory and results described here are largely consilient with three of the major psychological theories of autism, systemizing-empathizing bias (Baron-Cohen, 2009), enhanced perceptual function (Mottron et al., 2006), and the intense world (Markram and Markram, 2010), although they ground the patterns supporting each theory in a specific domain of human adaptation, intelligence. This compatibility of theories need not imply that autism has one or few specific causes at the genetic, neurological, and psychological levels—it has many—but it focuses attention on what information will be most useful to collect, to differentially diagnose the causes of autism for each specific individual. What alleles are related to what components of intelligence under the VPR model, and what is their overlap with alleles underlying different phenotypes found in autism? What neurological processes underlie negative associations between focal and diffuse attention, and verbal vs. rotational abilities (Johnson et al., 2008), and how do they relate to neurological differences among individuals with autism? More generally, what developmental and neurological components of intelligence are altered in autism, and how? And how can the genes, neurodevelopment, and psychology of each individual with autism, or subsets of individuals, be fit within these frameworks?

Fourth, comparisons of autism with schizophrenia for the genetic and phenotypic correlates of intelligence described here support the hypothesis that these two sets of conditions can be regarded as psychiatric, psychological, neurological and genetic “opposites,” especially as evidenced by consistent negative genetic correlations of schizophrenia risk with measures of intelligence. Jung (2014) indeed contrasts intelligence and the autism spectrum as diametric to creativity and the schizophrenia spectrum (see also Figure 1 in Crespi et al., 2016; Krapohl and Plomin, 2016), as two major, inversely-associated domains of human cognition. Inverse associations of intelligence with personality correlates of imagination (Openness and apophenia, defined as seeing pattern where none exists) are also supported by factor-analytic studies of personality structure, and by studies that relate working memory, white matter tract integrity, and dopaminergic neurotransmission to both intelligence and imagination (DeYoung et al., 2012). To the extent that autism represents most broadly a disorder of high intelligence (and low imagination), and schizophrenia a disorder of high imagination (and low intelligence), studying these psychiatric conditions will also provide novel insights into variation among neurotypical individuals, and human cognitive architecture, at their largest and smallest scales, with important implications for such fields as artificial intelligence and cognitive enhancement (e.g., Minzenberg et al., 2008; Blaser et al., 2014).

The primary limitations of the hypotheses and predictions evaluated here are that intelligence, as measured in most standardized tests, does not quantify aspects of social and emotional phenotypes that are also highly relevant to disorders such as autism and schizophrenia. Moreover, some key questions remain unresolved, such as how and why especially-high intelligence in one domain would tend to reduce intelligence test scores overall, how and why systemizing and empathizing are related to intelligence and its components (as well as genetic underpinnings), and how autism risk is mediated by polygenic effects, many of which apparently involve alleles for high intelligence, as well as by monogenic or oligogenic effects, which are expected to be deleterious and cause dysfunctions. Addressing these and other questions will require integration of data from evolutionary biology, genetics, the study of intelligence, and autism, and testing of hypotheses that involves spanning across these levels of analysis and theory.

## Author contributions

BC conceived and wrote the paper.

## Funding

I am grateful to NSERC for financial support.

### Conflict of interest statement

The author declares that the research was conducted in the absence of any commercial or financial relationships that could be construed as a potential conflict of interest.
